# Abnormal Paresthesias Associated With Radiofrequency Ablation of Lumbar Medial Branch Nerves: A Case Report

**DOI:** 10.7759/cureus.35176

**Published:** 2023-02-19

**Authors:** Aniroodh T Reddy, Nitin Goyal, Matthew Cascio, Jack Leal, Kanwardeep Singh

**Affiliations:** 1 Department of Physical Medicine & Rehabilitation, Nassau University Medical Center, East Meadow, USA; 2 Department of Anesthesiology, University of Toledo College of Medicine & Life Sciences, Toledo, USA

**Keywords:** interventional pain procedures, facet joint pain, back pain, chronic pain, rfa, paresthesia

## Abstract

Radiofrequency ablation (RFA) is an effective treatment that has occasionally been associated with transient paresthesias. This case report details an unusual presentation of paresthesias after lumbar medial branch RFA. A 48-year-old female patient reported pain, numbness, and swelling on the left buttock and posterolateral thigh. A physical exam revealed allodynia over the left posterolateral thigh without neurologic deficits two weeks after RFA of the left-sided lumbar medial branch nerves innervating the L4-L5 and L5-S1 facet joints. Shortly after the RFA of the contralateral targets, the patient complained of numbness of the right-sided lower back extending laterally from the right hip to the right knee. Imaging confirmed the appropriate placement of all needles in both procedures. Both instances of paresthesias resolved over time. This case report aims to demonstrate that RFA can be associated with unusual paresthesias and that these adverse effects do not warrant excessive workup.

## Introduction

Chronic pain is a complex disease that causes considerable personal distress to an estimated 84% of American adults at some point in their lifetime, resulting in more than $100 billion in annual expenses [1]. It is considered the leading cause of disability in the United States, with back and neck pain being the most common pain source. Facet joint arthritis is one of the most frequently encountered pathologies that cause axial back and neck pain [2]. The facet joint is the junction of the inferior articulating process of a vertebra and the superior articular process (SAP) of the vertebrae below. This joint is innervated by the medial branches from the dorsal rami of spinal roots at the level and one level above. These branches are found over the lateral border of the SAP [1]. Many patients with facet joint degeneration report that the pain is not controlled with conservative measures such as non-steroidal anti-inflammatory drugs and physical therapy and needs more aggressive treatment [2]. While opioid therapy can treat chronic pain, it is not ideal because of its adverse effect profile. To reduce opiate use, many providers elect to perform interventional procedures to reduce pain levels [2]. One of these procedures is a radiofrequency ablation (RFA) of the medial branch nerves that provides sensory innervation to the target facet joints [3]. The procedure involves a radiofrequency current through a nociceptive pathway to ablate the nerves that transmit pain impulses. An insulated electrode with a non-insulated tip is advanced towards the junction of the SAP and transverse process. A radiofrequency energy impulse is generated to produce a lesion via coagulative necrosis [1]. Providers will perform motor and sensory stimulation before ablation to ensure they do not inadvertently ablate the incorrect nerves. A systematic review of randomized controlled trials (RCTs) supported RFA as an efficacious therapy and safe in facet joint dysfunction compared to placebo [4]. Adverse events from the ablation of lumbar medial branches are uncommon but can include transient postoperative pain or paresthesias [1,5].

This case report demonstrates a patient who presented with paresthesias after bilateral RFA of lumbar targets that were appropriately tested with sensory and motor stimulation. This case report was previously presented as a poster at the Association of Regional Anesthesia’s Acute Pain Medicine & Regional Anesthesia’s Spring 2022 conference on April 1st, 2022. This case report was also previously presented as a virtual poster at the American Academy of Physical Medicine & Rehabilitation’s 2022 Annual Assembly on October 22nd, 2022. This case report was also previously presented as a poster at the Southeast Michigan Regional Research Symposium on June 1st, 2022.

## Case presentation

A 48-year-old female with a past medical history relevant for lumbosacral spondylosis with radiculopathy, right hip osteoarthritis, and stroke initially presented with chronic lumbar pain radiating down the right lower extremity to the calf with associated weakness, hemiparesis, and numbness for over six months.

**Figure 1:**
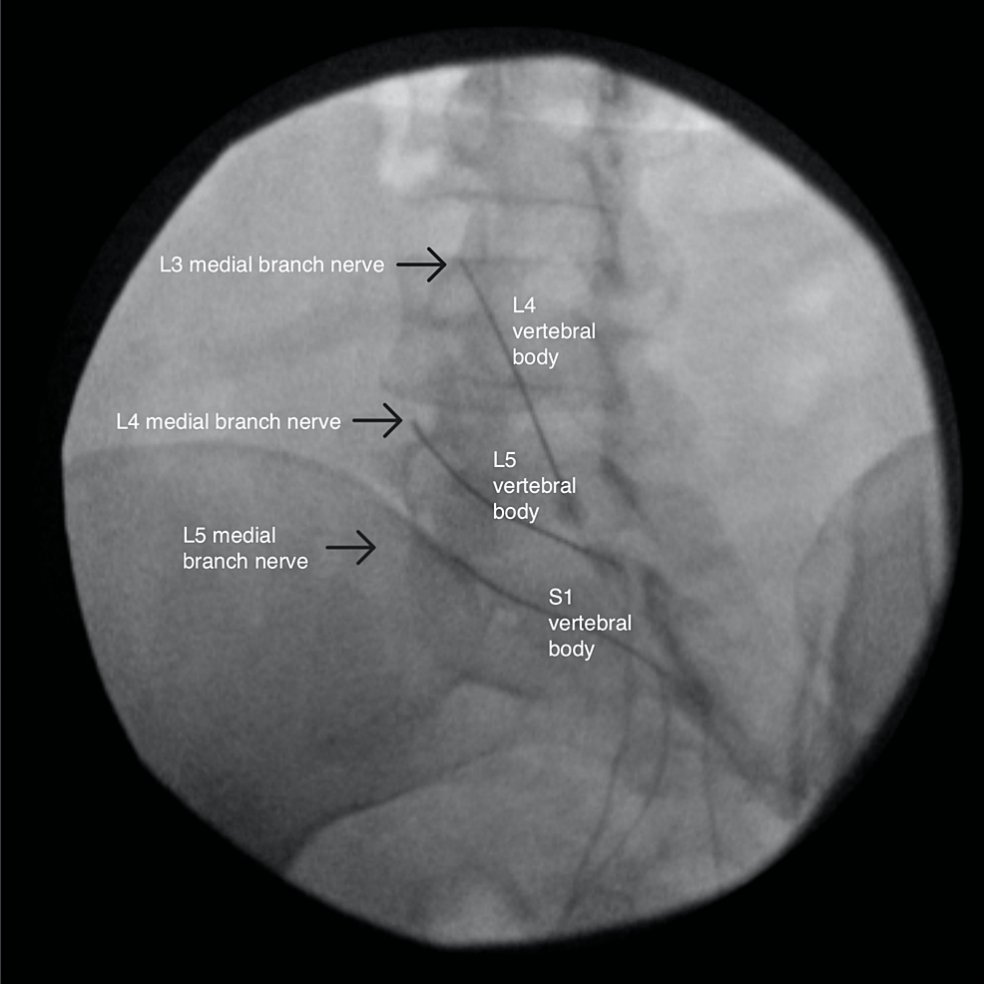
Oblique view of the left-sided radiofrequency ablation

**Figure 2:**
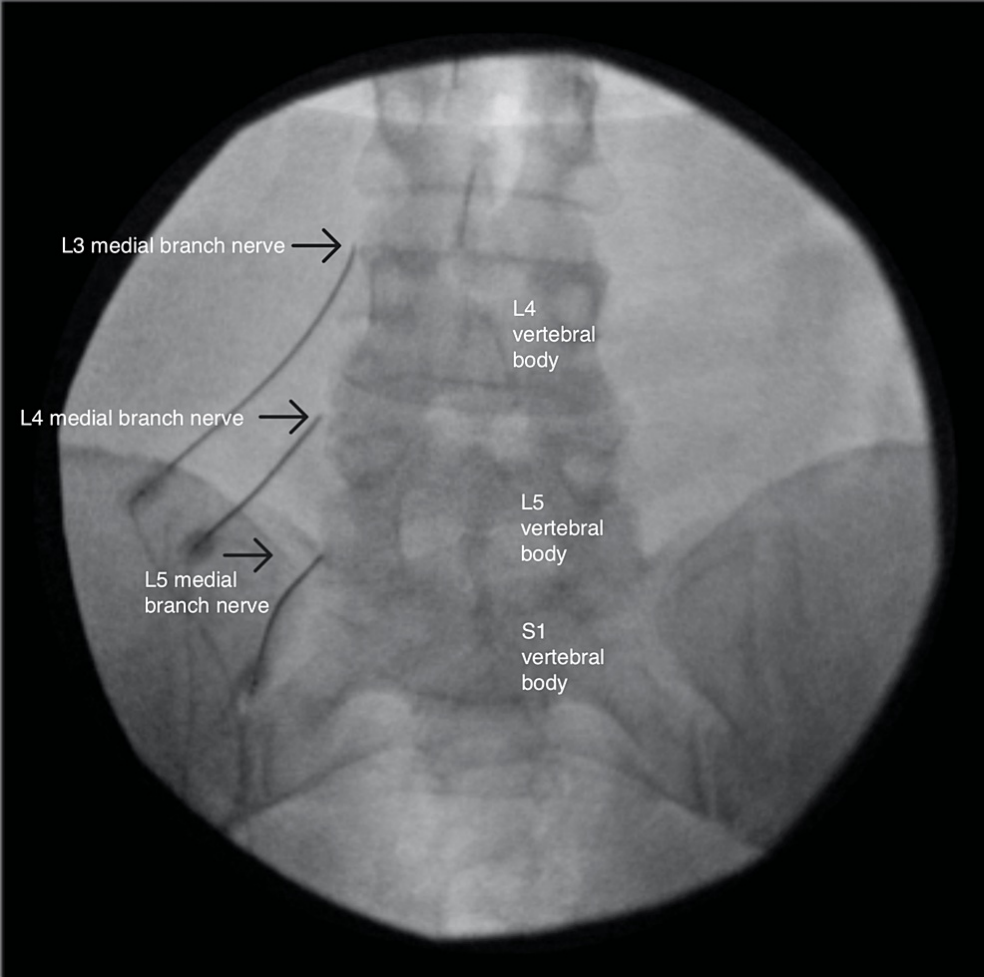
Anterior-posterior view of the left-sided radiofrequency ablation

**Figure 3:**
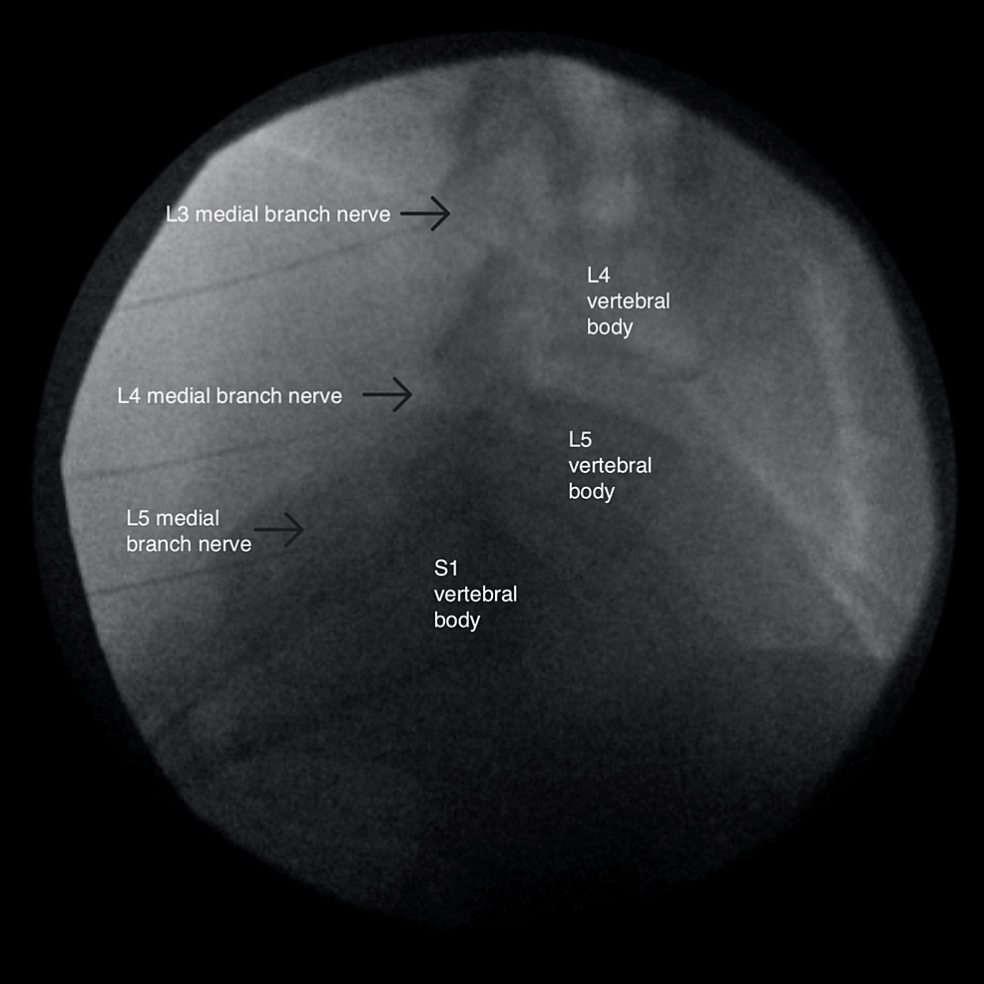
Lateral view of the left-sided radiofrequency ablation

At the onset of the pain, she reported waking up and suddenly having pain while standing. She was diagnosed with a 40% tear in her gluteus medius and right hip osteoarthritis. Conservative treatment with meloxicam, cyclobenzaprine, and physical and aquatic therapy provided relief of radicular pain but no improvement of axial back pain. She later suffered from a stroke, resulting in residual weakness throughout the right lower extremity. MRI of the lumbar spine showed bilateral L4-L5 facet hypertrophy without disc herniation or foraminal stenosis. Her physical exam revealed tenderness over the facet joints at the L4-L5 and L5-S1 levels and a positive right seated straight leg test. She was diagnosed with lumbosacral spondylosis and underwent diagnostic medial branch blocks (MBB) of the bilateral L4-L5 and L5-S1 facet joints. She reported 100% and 75% pain relief on the first and second MBBs, respectively. With the successful diagnostic blocks, she proceeded with RFA of the medial branches innervating the bilateral L4-L5 and L5-S1 facet joints, starting with the left side (Figures 1-3).

The RFAs were performed separately for time and cost efficiency. The RFA was performed with 15cm long, 20-gauge, 10mm-active tip, curved needles. Before ablation occurred, sensory and motor stimulation was performed, 2mL of 0.25% Bupivacaine was injected at each site, and repeat fluoroscopic imaging was taken to ensure accurate needle placement. The ablation was performed at 80 degrees Celsius for 90 seconds.

**Figure 4:**
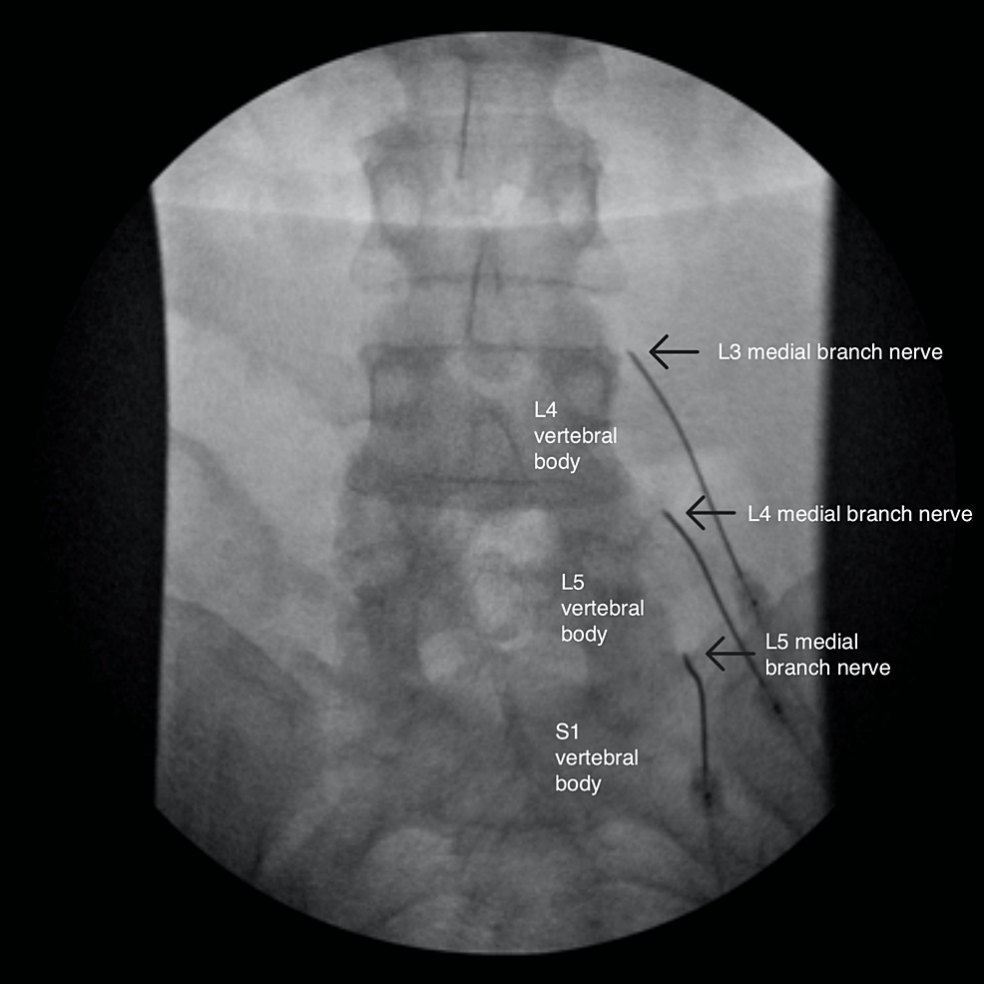
Anterior-posterior view of the right-sided radiofrequency ablation

**Figure 5:**
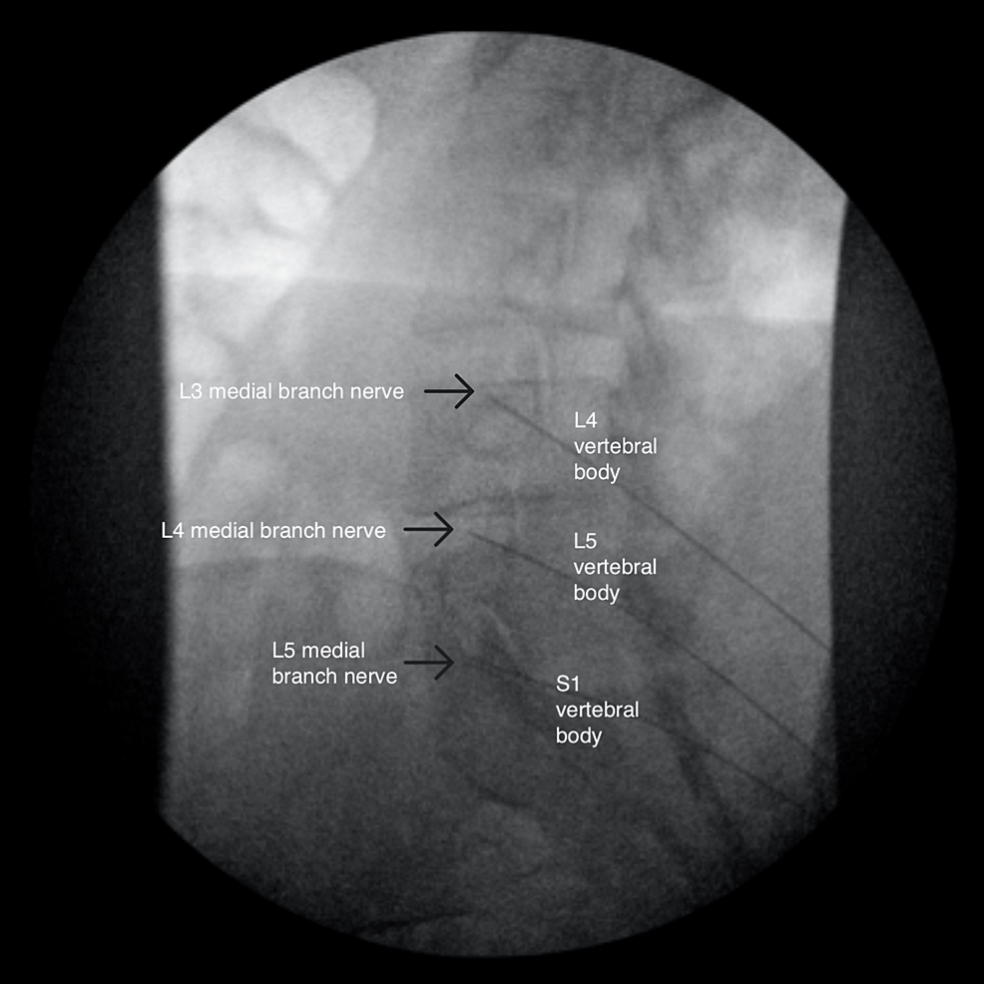
Oblique view of the right-sided radiofrequency ablation

**Figure 6:**
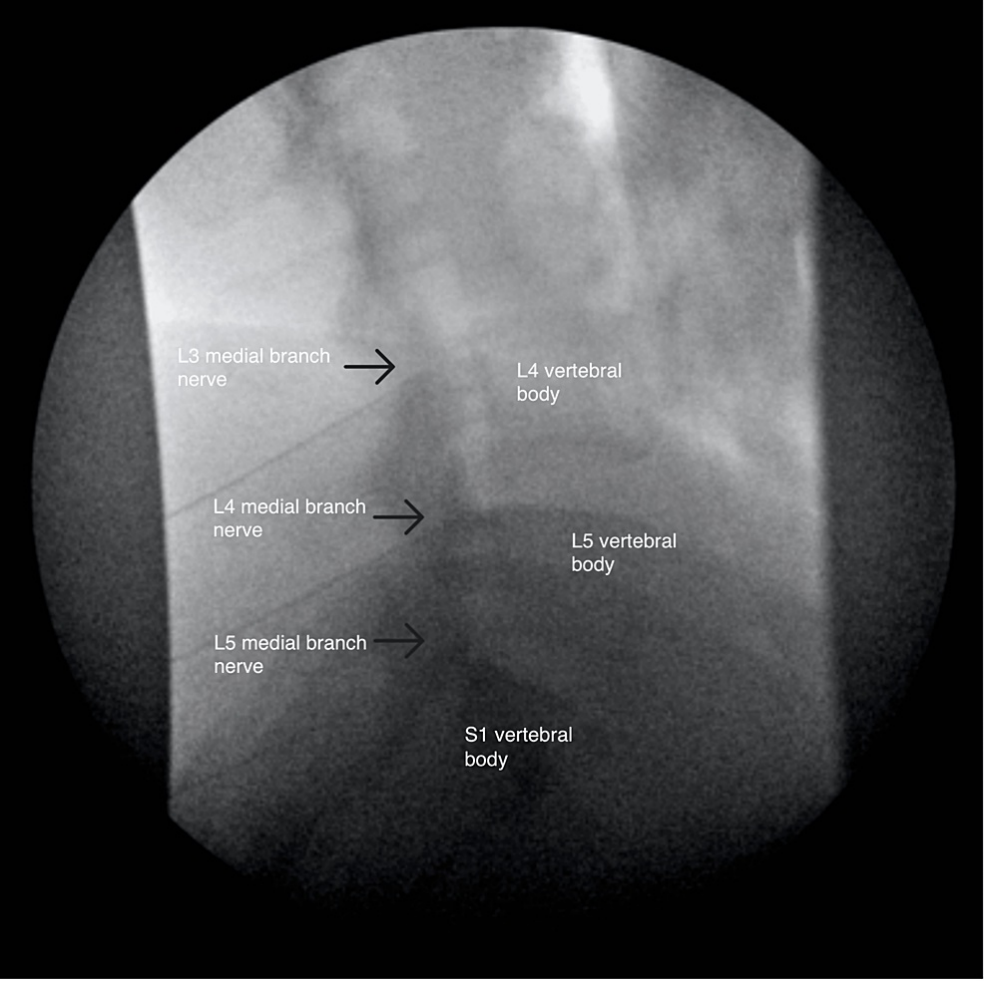
Lateral view of the right-sided radiofrequency ablation

After completing the left-sided RFA with appropriate sensory and motor stimulation testing, she initially reported a 100% improvement on the left. Two weeks later, the patient complained of pain and numbness on the left buttock and posterolateral thigh, with associated swelling. On physical exam, she had allodynia over the left posterolateral thigh with no neurologic deficits below the mid-thigh. Suspecting deafferentation pain secondary to peripheral neuropathy, she was prescribed lidocaine cream to alleviate the discomfort to minimal benefit. Later that day, the right-sided RFA was performed similarly with sensory and motor stimulation testing (Figures 4-6).

After one week, the patient complained of numbness of the right-sided lower back extending laterally from the right lateral hip to the right knee. One month after her RFA procedures, she reported her new radiating pain symptoms had gradually resolved. On review of imaging from both procedures, needle positioning was appropriately outside the neural foramen in anteroposterior, lateral, and oblique views. However, her facet pain improved by only 50% over two weeks and then gradually returned to baseline.

## Discussion

This case report presents a unique complication of RFA of the medial branch nerves innervating the facet joint. Ablations of the medial branches are normally performed after diagnostic medial branch blocks confirm temporary relief from the local anesthetic. The RFA is expected to produce longer relief of pain by destroying the medial branch nerves innervating the target facet joints [6]. A double-blinded randomized controlled trial demonstrated that this procedure’s efficacy is superior to placebo [7]. Systematic sets of guidelines guide practitioners on indications for these procedures [8]. Ablations can occur through several different methods, including thermal and chemical ablation via phenol or alcohol [9]. In this case, the nerves were thermally ablated. Thermal nerve ablation is a minimally invasive, relatively low-risk procedure [5,9]. Before the burn is completed, providers will perform motor and sensory stimulation to ensure that they do not inadvertently burn the incorrect nerves. If testing reveals inappropriate stimulation, the RFA needles are repositioned and retested. While low risk, the process of thermal ablation may lead to burns, worsened pain, or serious neurologic complications if proper placement and testing are not performed [10]. The most common postoperative complications include paresthesias, dysesthesias, and transient neuropathic pain [1]. In a retrospective analysis of 616 radiofrequency lesions, Kornick et al. reported that paresthesia occurred in 1.0% of the ablations. Of those six cases, three were characterized by localized pain lasting >2 weeks, and three involved neuritic pain lasting <2 weeks [11]. Our patient’s paresthesias of allodynia began two weeks after the left-sided RFA. At the time, because of the unique distribution of sensory numbness, it was theorized that there was an inadvertent ablation of an anatomical variant of sensory nerves. These could include the posterior cutaneous nerve of the thigh, posterior cutaneous nerve of the lateral femoral cutaneous nerve, or the perforating cutaneous nerve. About four days after the right-sided RFA, the patient had a similar constellation of symptoms, developing paresthesias around the right lateral hip that extended to the right knee. The patient’s paresthesias can be deemed unusual because the abnormalities began days to weeks after the procedure, persisted for multiple weeks, and occurred after both RFAs, which were performed separately on different days. However, both of the paresthesias did resolve eventually. This case report was written to establish that these side effects are possible with RFA, and with appropriate workup, should not raise concern for more significant pathologies.

## Conclusions

This case discusses abnormal paresthesia associated with radiofrequency ablations. Literature review reveals that, while paresthesias are common after RFAs, this patient had unusual paresthesia because they began days to weeks after each separately performed procedure. Furthermore, while these paresthesias occurred ipsilateral to the medial branch block, it should be noted that the ipsilateral nerves anastomose with their contralateral counterparts. Therefore, similar paresthesias could also be noted contralateral to the side of the procedure. The purpose of this case report was to demonstrate that RFAs may cause transient paresthesia of abnormal duration, onset, and duplicability. Practitioners should be prepared for post-procedure paresthesia for similar future cases and should not be overly concerned if an appropriate neurologic workup is completed.
